# The effect of a germline mutation in the APC gene on β-catenin in human embryonic stem cells

**DOI:** 10.1186/s12885-016-2809-9

**Published:** 2016-12-23

**Authors:** Nofar Yedid, Yael Kalma, Mira Malcov, Ami Amit, Revital Kariv, Michal Caspi, Rina Rosin-Arbesfeld, Dalit Ben-Yosef

**Affiliations:** 1 Wolfe PGD-Stem Cell Lab, Racine IVF Unit, Lis Maternity Hospital, Tel-Aviv Sourasky Medical Center, Tel Aviv, Israel; 2 Department of Cell and Developmental Biology, Sackler Faculty of Medicine, Tel-Aviv University, Tel Aviv, Israel; 3 Department of Clinical Microbiology and Immunology, Tel-Aviv University, Tel Aviv, Israel; 4 Departmant of Gastroenterology, Tel-Aviv Sourasky Medical Center, Tel Aviv, Israel

**Keywords:** Human embryonic stem cells (hESCs), Familial adenomatous polyposis (FAP), Adenomatous polyposis coli (APC), Cancer

## Abstract

**Background:**

Most cases of colorectal cancer (CRC) are initiated by inactivation mutations in the APC gene, which is a negative regulator of the Wnt-β-catenin pathway. Patients with familial adenomatous polyposis (FAP) inherit a germline mutation in one APC allele, and loss of the second allele leads to the development of polyps that will turn malignant if not removed. It is not fully understood which molecular mechanisms are activated by APC loss and when the loss of the second APC allele occurs.

**Methods:**

Two FAP human embryonic stem cell (hESCs) lines were derived from APC mutated embryos following pre-implantation genetic diagnosis (PGD) for FAP. These FAP-hESCs were cultured in vitro and following extended culture: 1) β-catenin expression was analyzed by Western blot analysis; 2) Wnt-β-catenin/TCF-mediated transcription luciferase assay was performed; 3) cellular localization of β-catenin was evaluated by immunoflorecence confocal microscopy; and 4) DNA sequencing of the APC gene was performed.

**Results:**

We have established a novel human in-vitro model for studying malignant transformation, using hESCs that carry a germline mutation in the APC gene following PGD for FAP. Extended culturing of FAP1 hESCs led to activation of the Wnt signaling pathway, as demonstrated by enhanced β-catenin/TCF-mediated activity. Additionally, β-catenin showed a distinct perinuclear distribution in most (91 %) of the FAP1 hESCs high passage colonies. DNA sequencing of the whole gene detected several polymorphisms in FAP1 hESCs, however, no somatic mutations were discovered in the APC gene. On the other hand, no changes in β-catenin were detected in the FAP2 hESCs, demonstrating the natural diversity of the human FAP population.

**Conclusions:**

Our results describe the establishment of novel hESC lines from FAP patients with a predisposition for cancer mutation. These cells can be maintained in culture for long periods of time and may serve as a platform for studying the initial molecular and cellular changes that occur during early stages of malignant transformation.

**Electronic supplementary material:**

The online version of this article (doi:10.1186/s12885-016-2809-9) contains supplementary material, which is available to authorized users.

## Background

Colorectal cancer (CRC) is one of the leading causes of cancer-related mortality [1]. About 50 % of all CRC patients will develop metastases and ultimately die from the disease. Most CRC cases arise from two somatic unrelated events, however, approximately 5 % of CRCs are initiated by an inherited genetic mutation which inevitably leads to the acquisition of a second somatic mutation. In all cases, progression to carcinoma occurs through the accumulation of multiple somatic mutations, leading to malignant transformation and development of an invasive cancer [1–3].

One of the most critical genes mutated in CRC is the adenomatous polyposis coli (APC) tumor suppressor gene [1, 2]. APC encodes a large multi-functional protein [4], and its main role in tumorigenesis lies in its ability to negatively regulate Wnt signaling by controlling cellular levels of β-catenin [1]. Wnt signalling is a key developmental pathway involved in embryonic development, cell differentiation, cell proliferation and tissue maintenance in adults [5, 6]. However, the aberrant constitutive activation of the Wnt pathway that is caused by APC mutations in many cases leads to uncontrolled cell proliferation and tumorigenic transformation, CRC being the most notable among them [6].

Since APC mutations are detected very early in the adenoma-carcinoma sequence, the APC protein has been suggested to act as a "gatekeeper" of colorectal carcinogenesis, which means that functional loss of APC is a prerequisite for the progression towards malignancy. Around 85 % of all sporadic and hereditary colorectal tumors show loss of APC function [1]. Individuals affected by familial adenomatous polyposis (FAP) carry a germline mutation in the APC gene ('first hit'), and show autosomal dominant inheritance with essentially 100 % penetrance (i.e., all will develop cancer [3, 7, 8]). Young FAP patients start to acquire additional mutations (somatic mutations or the 'second hit') in the second allele of the APC gene, leading to its functional loss and to the development of adenomatous colon polyps, which invariably progress to colon cancer if not removed.

The APC gene includes a mutation cluster region (MCR) which is prone to mutations. The cell will have a selective advantage for tumor formation when at least one of the mutations (germline or somatic) is located within the MCR region that includes multiple β-catenin binding sites. Indeed, APC mutations in colorectal tumors are distributed non-randomly within the gene [9], with the position and type of the somatic APC mutation depending on the germline mutation [9–16].

Most of our knowledge about the initiation and development of CRC came from studies performed in cancer cells derived from CRC-affected patients [17]. In addition to the APC mutation, these already differentiated cells reportedly carry some other mutations that are only partly characterized and thus have limitations in providing necessary data on the initial molecular steps leading to cancer formation. Another research model for CRC is genetically manipulated mice with different mutations in the APC gene. Although most of these APC mutations in mice are embryonically lethal, the severity of the cancer predisposition is variable [18]. Numerous APC genetically altered mice have been generated and serve as models for colon adenoma and cancer, but their phenotypes are different from the human disease [19]. For example, several genetic mouse models generate tumors predominantly in the small intestine, in contrast to human CRC, in which tumors are found in the colonic epithelium [20]. Carcinogen treatment of mice generates colonic neoplasia, but these mice show specific gene expression patterns that do not represent the entire development of human CRC [20]. Therefore, although these models are very important for studying colon carcinogenesis, they are inadequate for the study of the earliest molecular mechanisms underlying malignant transformation in humans.

Human embryonic stem cells (hESCs) have already been proven to be a valuable tool for studying human genetic disorders [21–25]. Pre-implantation genetic diagnosis (PGD), a procedure used to obviate the inheritance of mutations in affected families, has recently been established for FAP families as well. In the current study, we use two FAP hESC lines derived from embryos that inherited the APC germline mutation following PGD for FAP carriers (Lis25_FAP1 published in [26]). These cell lines, to the best of our knowledge, are available solely in our lab, and they comprise a valuable model for unraveling the very early mechanisms leading to malignant transformation in the colon.

## Methods

### Derivation and culture of hESC lines carrying APC mutations

The use of spare in-vitro fertilization (IVF)-derived embryos that have been diagnosed by PGD as genetically affected for the generation of hESCs was approved by the Israeli National Ethics Committee (7/04-043). FAP-affected embryos were cultured to the blastocyst stage. At day 6–7 of development, the embryos were micromanipulated to isolate the inner cell mass (ICM) cells for derivation of hESCs as previously described [27]. Isolated clumps of ICM cells were plated on mouse embryonic fibroblast cells (MEFs: feeder cell layer of mitomycin C–inactivated treated mouse embryonic fibroblasts) and cultured in hESC media (KnockOut Dulbecco's Modified Eagle Medium [KO-DMEM, by Gibco] supplemented with 20 % KO-serum replacement, 1 % nonessential amino acids, 1 mM l-glutamine, 0.5 % insulin transferrin–selenium, 50U/mL penicillin, 50 mg/mL streptomycin, 0.1 mM beta-mercaptoethanol, and 30 ng/mL bFGF). Outgrowth-containing cells were manually cut and propagated, resulting in a stable culture of undifferentiated hESCs as previously described [28].

Two FAP hESC lines were examined in this study: Lis25_FAP1 (FAP1) that we have already described elsewhere [26] and Lis30_FAP2 (FAP2) that we have recently derived. Three non-mutated APC hESC lines were used as controls: HUES64, HEFX1 and HUES-6 [29–31], The cells were cultured on mitomycin-C-treated MEFs in hESC medium. Characterization of hESCs included expression of *OCT4*, *SSEA-4* and *TRA-1-60* by immunofluorescence. FACS analysis of undifferentiated hESCs was performed using Alexa Flour-488 SSEA-3 antibodies (BioLegend) and their respective isotype controls. Samples were analyzed using a BD FACS Canto flow cytometer (BD Biosciences).

Karyotype analysis was performed as previously described [22]. The differentiation potential was assessed by teratoma induction, as previously described [22], and teratoma sections were stained with eosin and hematoxylin.

### Immunofluorescence

FAP1, FAP2 and normal hESC lines were fixed, washed with PBS, permeabilized with PBS containing 0.1 % Triton (PBT) and blocked in 1 % BSA and 0.1 % Triton in PBS for one hour. The cells were then incubated at room temperature with primary antibodies (rabbit anti-β-catenin, Santa Cruz Biotechnology; mouse anti-Rab11A, Abcam; mouse anti-TRA-1-60 Santa Cruz Biotechnology; mouse anti-OCT-3/4, Santa Cruz Biotechnology; mouse anti-SSEA-4, Santa Cruz Biotechnology) and further incubated with secondary antibodies (goat anti-rabbit and donkey anti-mouse, Invitrogen). The cell nuclei were stained with 5 μg/ml 4′,6-diamidino-2-phenylindole (DAPI, Sigma) or with 5 μM 1,5-bis (2-(di-methylamino)ethylamino)-4,8-dihydroxyanthracene-9,10-dione (DRAQ5, Cell Signaling). The slides were visualized by confocal microscopy or by phase contrast microscopy (Leica SP5, Leica Microsystems, Bannockburn, IL).

### Western blot analysis

Protein was extracted from hESCs grown on matrigel (1:100 in KO-DMEM), using 100 μl lysis bufferX1 (Promega) with a 1 % protease inhibitor cocktail (Sigma). Cell lysates were incubated for 20 min on ice, centrifuged, and the supernatants were separated on 7.5 % SDS-polyacrylamide gel electrophoresis (SDS-PAGE), followed by transfer to nitrocellulose membranes (0.2 μm, BIO-RAD) using BIO-RAD Mini Trans-Blot Cell. The membranes with the proteins were subjected to blocking solution (0.001 % TWEEN-20 in phosphate buffered solution (PBS) with 5 % low fat milk, Sigma). They were then incubated with primary antibody overnight at 4 °C, and washed with 0.001 % TWEEN-20 in PBS, followed by incubation for 1 h at room temperature with horseradish peroxidase-conjugated secondary antibody. After washing, the membranes were exposed to enhanced chemiluminescence detection analysis (EZ-ECL, Biological Industries). The antibodies used were: rabbit anti β-catenin, Santa Cruz Biotechnology; mouse anti-β-actin, Abcam; peroxidase-conjugated goat anti-rabbit and peroxidase-conjugated goat anti-mouse, Jackson Immune Research.

### Luciferase reporter gene assay

Transfection of undifferentiated hESCs was carried out by a jetPRIME® transfection kit (Polyplus) following the manufacturer's instructions. The cells were seeded on 24-well plates, cover with matrigel (1:100 in KO-DMEM,) and grown to 60–80 % confluence. Transfection was carried out with 0.6 μg of DNA (pGL3-OT (pTOPFLASH) or pGL3-OF (pFOPFLASH) luciferase reporter constructs containing three copies of either wild-type (WT) or mutated TCF binding element, respectively, and a *Renilla* Luciferase Reporter Vectors, to monitor transfection efficiency, mixed with 1.2 μl jetPRIME reagent for 4 h incubation, and then replaced by fresh growth medium. The cells were harvested on ice 48 h later by reporter lysis buffer (Promega) and their luciferase activity was measured by Lumistar Optima (BMG LABTECH) following the manufacturer's instructions. The statistical analysis was performed by Welch's *t* test. A *p* value of 0.05 was considered significant.

### Single-cell PCR for analyzing APC mutations in FAP patients

The partners of couples that underwent IVF treatment for the purpose of PGD of which one of them is a carrier of a pathogenic mutation in the APC gene and had severely affected relatives or aborted fetuses with FAP. At day 3 post-fertilization, embryos at the 6–8 cell stage were biopsied by the aspiration of 1–2 blastomeres from each. The biopsied blastomeres were then subjected to single-cell genetic analysis for the specific APC mutation as well as for 3–6 polymorphic markers flanking the mutation (as described by us for other monogenic diseases [32]. Multiplex nested PCR was performed in order to analyze the mutation and the flanking polymorphic markers. Normal and mutated PCR products were differentially identified by restriction enzymes: NruI for analyzing the R332X mutation in FAP1 hESCs, and BstNI for analyzing IVS14 + 1 G > A mutation in FAP2. Both enzymes recognized and cleaved only the WT alleles.

Informative polymorphic markers were analyzed using Gene Scan (ABI 3130XL Genetic Analyzer). According to that analysis, embryos diagnosed as carrying the normal allele were transferred back to the uterus to allow implantation and the development of a pregnancy with a healthy fetus. Embryos diagnosed as inheriting the germline APC mutation were donated by the couple for the derivation of hESCs after they signed informed consent for the use of spare PGD embryos for the generation of hESCs, a study approved by the Israeli National Ethics Committee (7/04-043) [27]. A protocol similar to the one described above for single-cell PGD analysis was also used to confirm the inheritance of the mutation in the established FAP hESC lines.

### Sequencing of the APC gene

DNA was extracted from hESC lines following culture on matrigel (1:100 in KO-DMEM) using the QIAGEN-flexigene DNA kit (Cat # 51204, QIAGEN). DNA extraction was performed according to the manufacturer’s protocol. Amplification of hESCs DNA was performed encompassing the MCR region, the APC gene "hot spots" and the germline mutation. The PCR products were purified by the QIAquick PCR purification kit (QIAGEN) following the manufacturer's instructions, and sequenced using the ABI330XL (Sequencer ABI, Center of the Life Sciences Faculty in Tel Aviv University).

The entire APC gene was also sequenced using Pronto Diagnostics kit specifically aimed at sequencing all the coding sequences of the APC gene. The extracted DNA was sent to ProntoLab™, Pronto Diagnostics' molecular services laboratory (Tel Aviv, Israel) which used Multiplicom's (Neil, Belgium) FAP MASTR™ and MID Dx 1–48 for Illumina MiSeq® kits for library preparation. Illumina’s (San Francisco, CA) MiSeq Reagent Kit v2 (500 cycle) was used to run the library on the MiSeq instrument. The FAP MASTR™ kit enables identification of point mutations by complete coverage of all coding sequences of the APC gene. Data analysis was carried out using SeqNext module v4.1.2 of the Sequence Pilot software (JSI medical systems, Kippenheim, Germany).

## Results and Discussion

### Derivation of FAP-hESC lines with different mutations in the APC gene

**Fig. 1:**
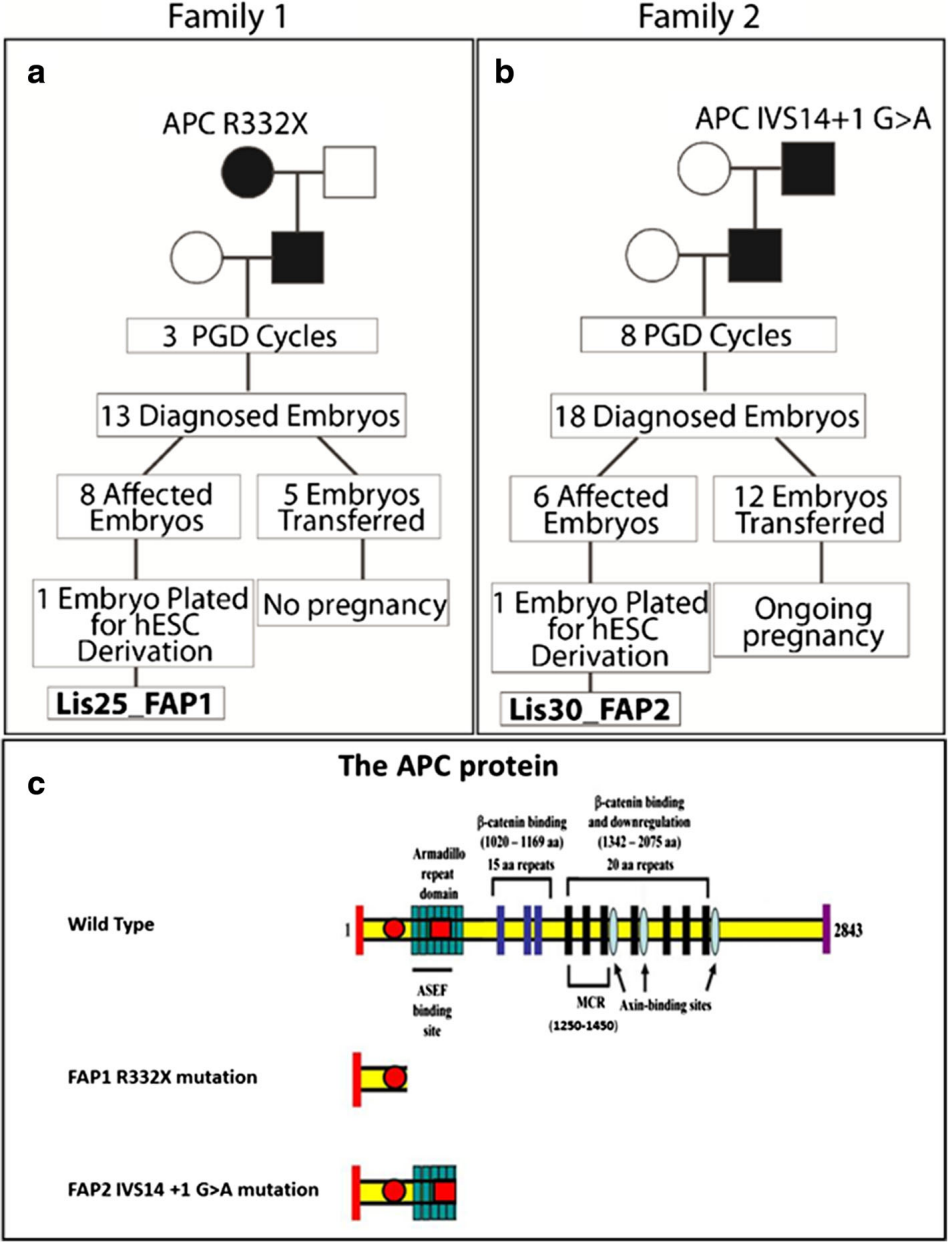
A family tree for couples with FAP who donated their APC-mutated embryos for the derivation of FAP hESC lines. **a**, **b** Two couples with different APC mutations for FAP who underwent PGD. The Lis25_FAP1 line carries the APC mutation R332X (**a**), and the Lis30_FAP2 line carries the APC mutation IVS14 + 1 G > A (**b**). **c** The APC protein with the localization of the germline mutations (*red* circle for FAP1 R332X mutation and *red* square for FAP2 IVS14 + 1 G > A mutation)

**Fig. 2:**
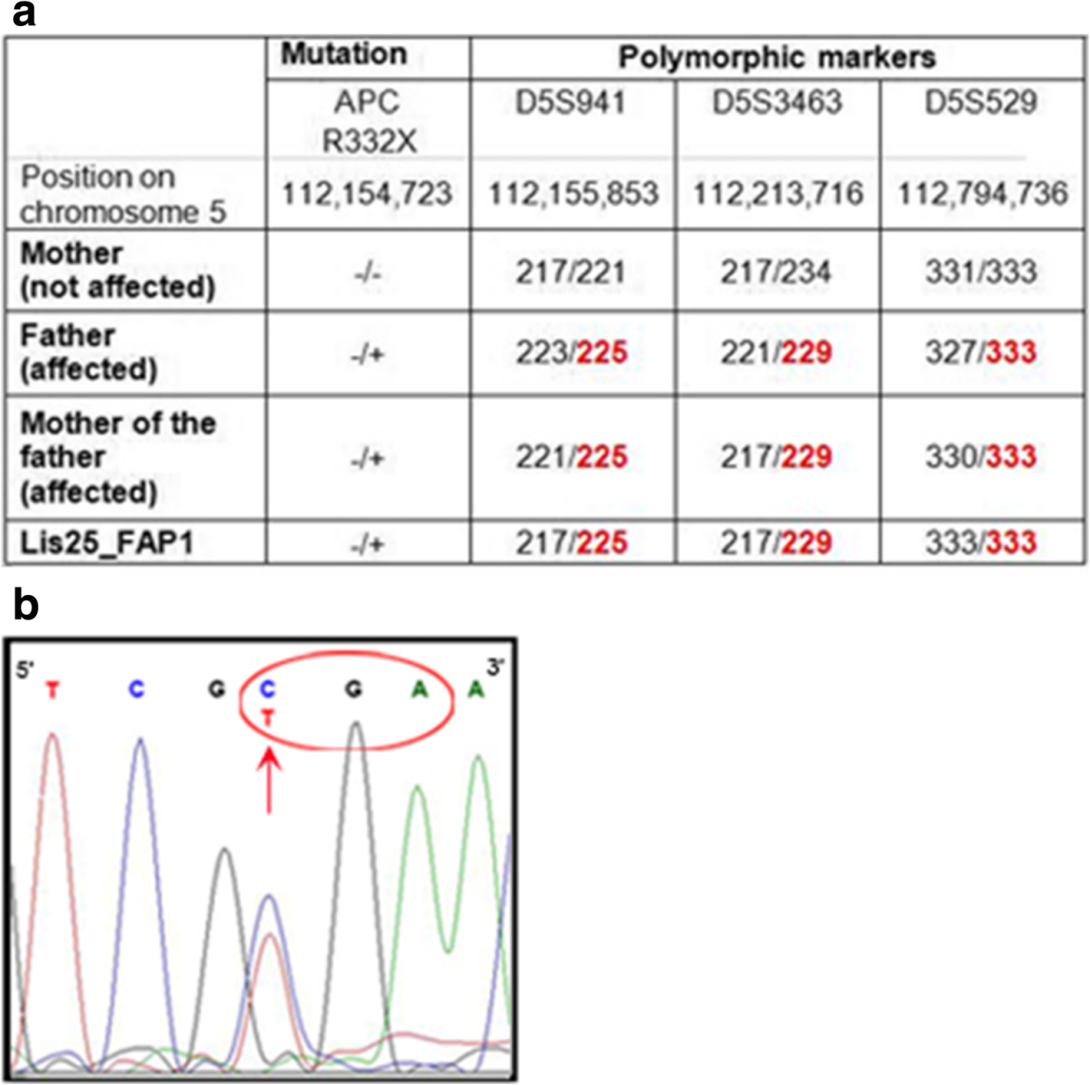
Confirmation of the APC germline mutation in FAP1 hESCs. **a** The mutation (R332X) and 3 polymorphic markers flanking the mutation area (D5S941, D5S3463, D5S529) for showing that the FAP1 cells, indeed, inherited the mutated APC allele from the affected father (mutated allele in *red*, normal allele in *black*). **b** DNA sequencing of the mutation area showing a thymine (*red* arrow, R332X mutation) instead of cytosine (normal allele) and resulting in a stop codon

The Lis25_FAP1 and Lis30_FAP2 hESC lines were established following PGD for FAP patients by means of approved protocols [26, 27, 33]. All the data on PGD cycles and the number of diagnosed embryos for each FAP family are illustrated in Fig. 1. Lis25_FAP1 (FAP1) was derived from Family 1, in which the father inherited the APC R332X mutation from his mother (Fig. 1a). This couple underwent 3 PGD cycles in which a total of 13 embryos were diagnosed. Eight affected embryos were donated for hESC derivation, of which one was plated and the Lis25_FAP1 hESC line was established. The APC R332X mutation led to the expression of a truncated APC protein. The Lis30_FAP2 (FAP2) was derived from Family 2, in which the father inherited the mutation from his father. This couple underwent 8 PGD cycles in which a total of 18 embryos were diagnosed. Six affected embryos were donated for HESCs derivation, of which one was plated and the FAP2 hESC line was established (Fig. 1b). It carried the germline mutation IVS14 + 1G > A coding for a stop codon in the first nucleotide of intron 14 which led to a splicing error that resulted in a truncated protein. The molecular structure of the APC protein with the localization of the germline mutations of the FAP1 and FAP2 hESC lines is shown in Fig. 1c. In order to confirm the inheritance of the mutated APC allele within the FAP hESCs, the specific sequence of the APC mutations, in addition to 3–6 polymorphic markers flanking the mutation, were analyzed with the same set of primers used for the single-cell PGD analysis (Fig. 2a; Additional file 1: Figure S1). The region around the germline mutation in FAP1 was also amplified and sequenced (Fig. 2b).

### Characterization of FAP hESC lines as pluripotent stem cells

**Fig. 3:**
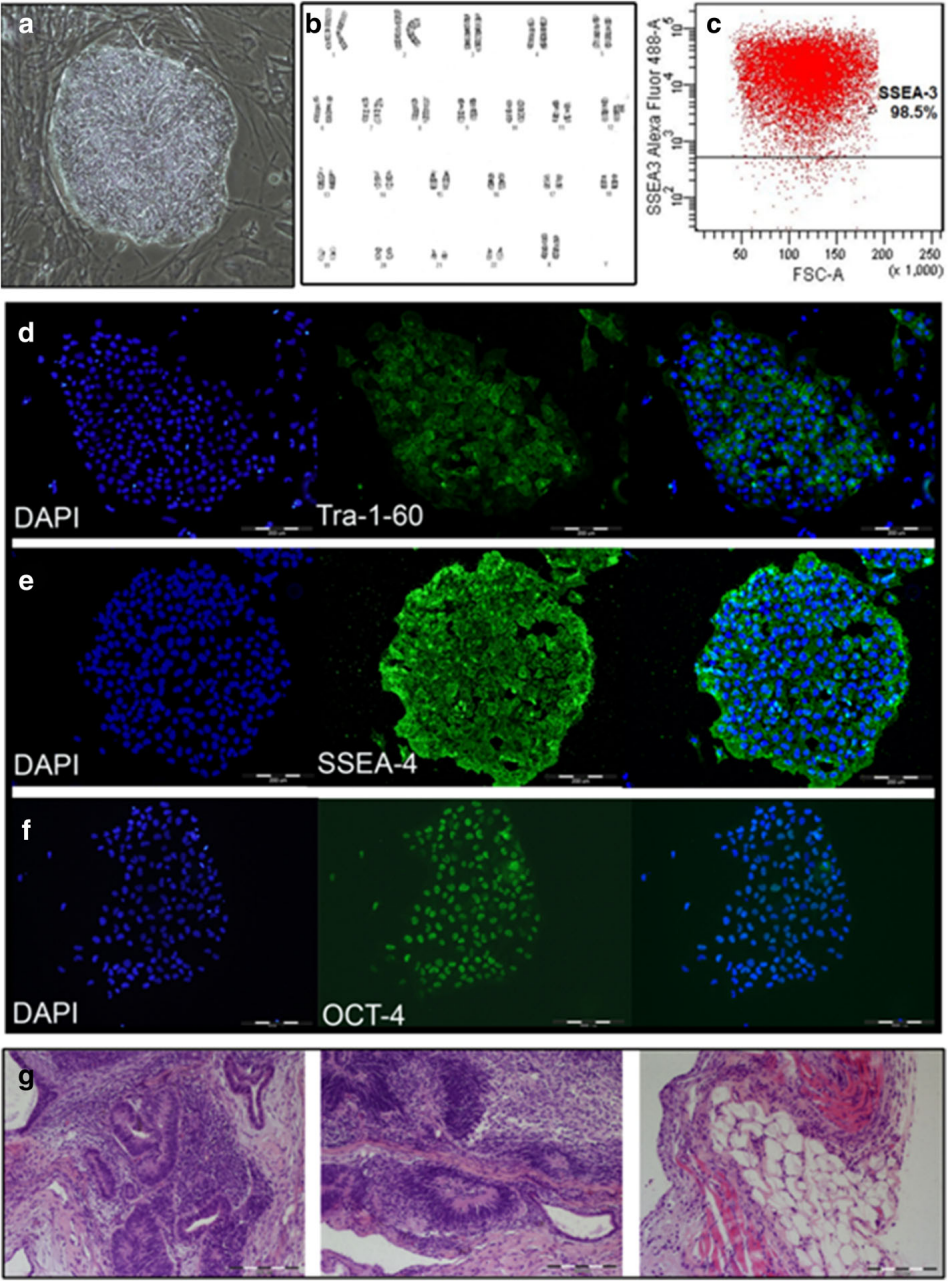
Characterization of a FAP1 hESC line. **a** A colony of FAP1 cells at p54 with a typical morphology of hESCs. **b** Karyotype analysis. **c** FACS analysis for the pluripotent marker SSEA3. **d**-**f** Immunostaining for the pluripotent markers (*green*) Tra-1-60 (**d**), SSEA-4 (**e**), OCT4 (**f**), and the nuclear marker DAPI (*blue*) and their overlay. **g** Hematoxylin and eosin staining of 8-week-old FAP1-derived teratoma sections showing cell different structures of differentiated cells

The two FAP hESC lines (FAP1 and FAP2) were propagated for a long period of time (>50 passages) in culture without losing their pluripotent properties. Both lines demonstrated a typical morphology of hESCs with a normal karyotype (Fig. 3; Additional file 2: Figure S2). Using FACS analysis for quantifying the percent of undifferentiated pluripotent cells, we demonstrated that approximately 90 % of the FAP1 and FAP2 cells were pluripotent (Fig. 3c, Additional file 2: Figure S2C). Immunofluorescence analysis demonstrated that FAP hESCs expressed a panel of markers which are specific for undifferentiated cells, including the surface markers Tra-1-60, SSEA-4 and the nuclear marker OCT4 (Fig. 3d-f; Additional file 2: Figure S2D-F). Pluripotency of FAP1 hESCs was also confirmed by inducing teratomas following injection of the cells into immunodeficient mice. Hematoxylin and eosin staining of paraffin-embedded sections of FAP1 teratoma developed in mice revealed different structures of differentiated cells (brain like structure, adipose, skeleton muscle, endothelial progenitors etc.), indicating their pluripotency (Fig. 3g). Altogether, our results demonstrated that both FAP1and FAP2-hESCs lines inherited their parental mutations and remained pluripotent throughout all passages used for all experimental procedures.

### Analyzing the effect of extended culturing on FAP-hESCs and APC

Genetic and epigenetic instability have been strongly associated with various types of cancer. Extended culture of hESCs has already been shown to be associated with genetic instability [24, 25, 34–36], and some of the most frequent chromosomal changes observed in these cells, such as trisomies of chromosomes 12 and 17, are similar to those seen in malignant germ cell tumors [37, 38]. The FAP hESCs carry a mutation in one allele of the APC gene (the germline inherited mutation) that leads to a predisposition to cancer. Both APC alleles are either mutated or lost in most colonic adenomas. We therefore hypothesized that during extended culture, the hESCs will acquire additional mutations, some of them in the second APC allele, (a second APC ‘hit), which will result in complete loss of APC function and provide the cells with a selective advantage that will eventually dominate the whole population. In order to detect the acquisition of the second mutation, we tested the expression levels and activity of β-catenin as well as its subcellular localization, given that mutations in APC lead to accumulation and nuclear translocation of β-catenin in many cancers [39].

Undifferentiated FAP-HESCs were propagated for >45 passages and the cell extracts of high passages were compared to those of early passages. In addition, as APC is extremely difficult to detect in minute tissue samples, we assayed its most affected downstream counterpart β-catenin.

**Fig. 4:**
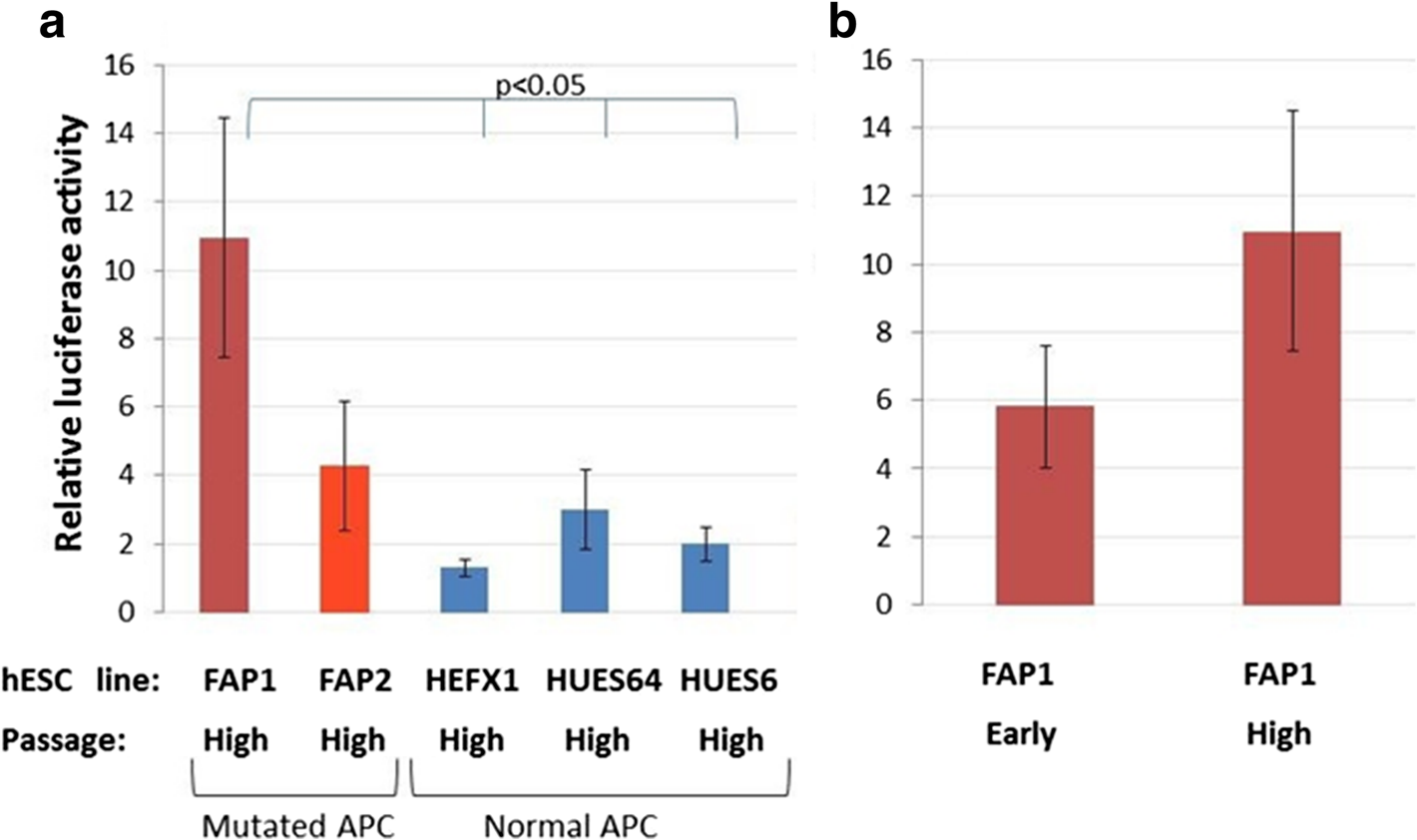
Wnt-β-catenin/TCF-mediated activity in hESC lines. **a** FAP1, FAP2 and normal APC hESC lines of high passage were transfected with pTOPFLASH or pFOPFLASH and Renilla (FAP1 passage 82, FAP2 passage 80, HEFX1 passage 45, HUES64 passage 48, HUES6 passage 53,), and their lysates were measured for luciferase activity. **b** FAP1 early (passage 12) and FAP1 high (passage 82) were measured for their luciferase activity. These data describe relative mean values (±STDV) of three independent experiments performed in duplicates. Welch's *t*-test between each one of the pairs was performed

Western blot analysis of protein extracted from early and high passages of FAP-hESCs cells as well as from control hESC lines with non-mutated APC (HEFX1, HUES64, HUES6-termed control) demonstrated wide fluctuations in β-catenin levels (Additional file 3: Figure S3). We therefore decided to perform a more sensitive assay that measures the activity of β-catenin, Wnt-β-catenin/TCF-mediated transcription luciferase assay. This assay is based on the accumulation of nuclear β-catenin which, in turn, binds T-cell factor (TCF)/lymphoid enhancer factor (LEF) to activate the transcription of Wnt target genes. In the absence of the degradation complex (e.g., a truncated APC protein resulting from a mutation in both alleles), the β-catenin-mediated signal should increase. Indeed, our results demonstrated that high passage FAP1 cells showed enhanced β-catenin/TCF-mediated activity compared to control cells (*P* < 0.05, Welch's *t* test) (Fig. 4a) and to early passage (p12) FAP1 cells (Fig. 4b). In contrast, luciferase activity of FAP2 was not significantly different from the control lines, even at high passage (Fig. 4a).

**Fig. 5:**
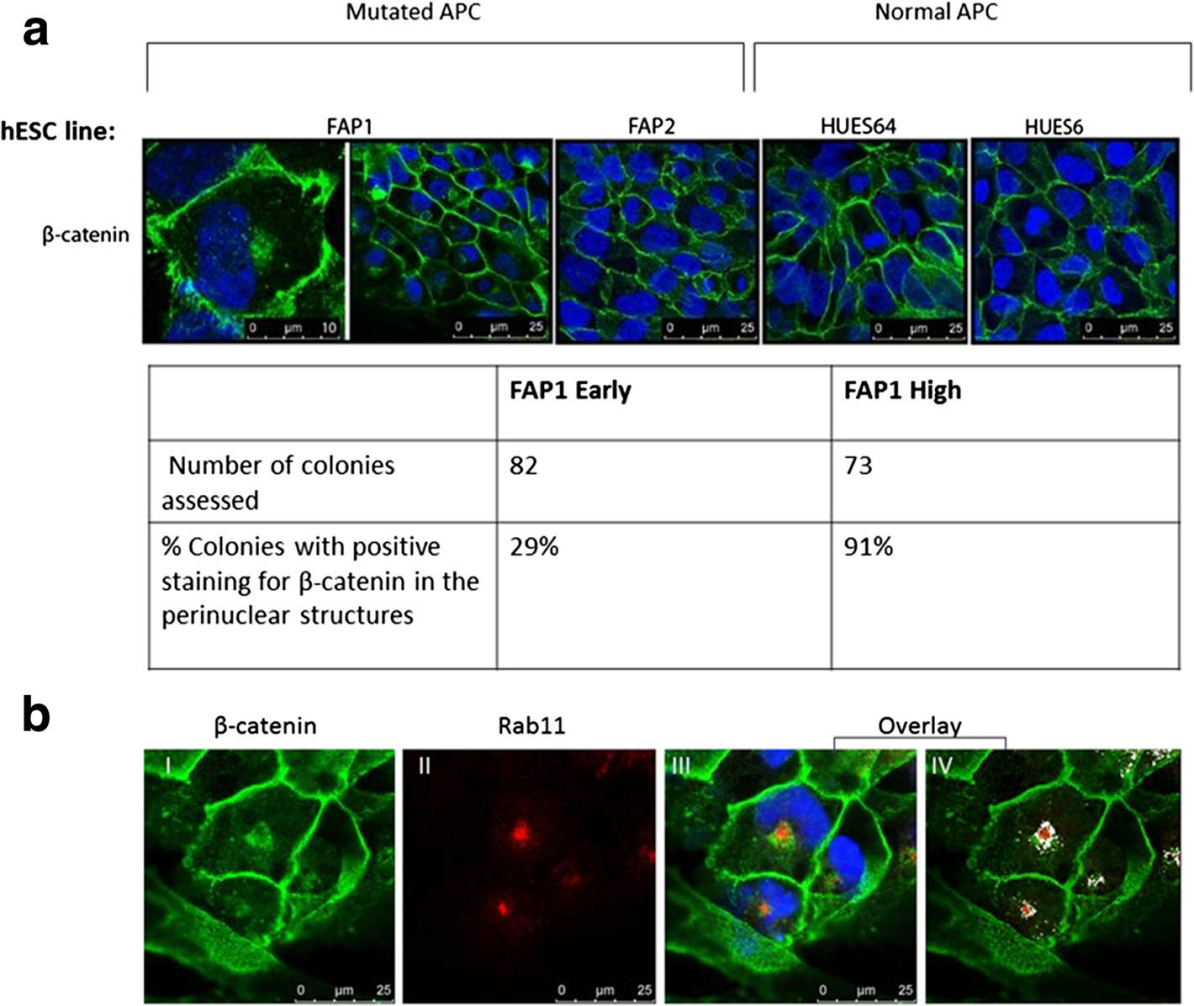
**a** Cellular localization of β-catenin in hESC lines. FAP1 hESCs, FAP2 hESCs and two normal APC hESC lines were stained with rabbit anti-β-catenin antibody followed by the secondary antibody Alexa Flour® 488 goat anti-rabbit (*green*). The cell nuclei were stained with DRAQ5 and examined with confocal microscopy. *Red* arrows show accumulation of β-catenin in the perinuclear structures in FAP1. Quantification table of β-catenin localization to the perinuclear structures. Only 29 % of FAP1 hESCs colonies from early passages (24/82; p 12) were stained positive for β-catenin next to the nucleus, while all the rest showed only membrane staining. In contrast, 91 % of the high passage colonies (67/73; p82) were stained positive for β-catenin next to the nucleus. **b** Colocalization of β-catenin and RAB11. The FAP1 hESC line stained with rabbit anti-β-catenin (I) and mouse anti-RAB11 (II) followed by the secondary antibodies Alexa Flour® 488 goat anti-rabbit (*green*) and Cye2 sheep anti-mouse (*red*). The cell nuclei were stained with DAPI and examined with confocal microscopy. An overlay picture is shown in III, and the colocalized area is marked in *white* (IV)

Inactive β-catenin is usually localized to the membrane or cytoplasm, however, upon activation it translocates and accumulates into the nucleus. We therefore analyzed β-catenin activation also by determining its cellular localization using an anti-β-catenin antibody and confocal laser microscopy. Interestingly, we found that β-catenin was localized to perinuclear structures in most high passage FAP1 hESCs (Fig. 5a). In contrast, β-catenin was localized solely in the membrane in the normal APC hESC lines and in the FAP2 hESCs. Quantification of the colonies in which β-catenin had a perinuclear localization demonstrated that β-catenin exhibited perinuclear localization in 91 % of the colonies in high passage of FAP1-hESCs, compared to only 29 % in the early-passage. Furthermore, a perinuclear localization pattern was detected in all of the colony cells in colonies in which β-catenin was perinuclear. These results indicated that extended culture induced changes in β-catenin sub-cellular localization, which may indicate the acquisition of a somatic mutation in the APC gene.

In view of the fact that β-catenin in high passage FAP1-hESCs is sequestered to yet unknown cellular structures next to the nucleus, we used RAB11 that was previously shown to co-localize with β-catenin in similar structures [40]. Our results demonstrated that, indeed, β-catenin (green, Fig. 5b) is localized to the same perinuclear structures as RAB11 in FAP1 cells (red, Fig. 5b). However, while RAB11 was concentrated mainly in the center of these structures, β-catenin staining was more diffused. It is possible that the perinuclear localization of β-catenin in FAP1 hESCs is an indication of regulatory disruption that resulted in different sub-cellular sequestering. These results together with the elevated Wnt-β-catenin/TCF activity may suggest a progressive process in which β-catenin migrates from the cell membrane to the cytosol due to the dismantling of the APC complex. β-catenin is then directed to the nuclear vicinity, where it interacts with a variety of proteins that stabilize it to the perinuclear compartment [41]. A similar sub-cellular localization of β-catenin was observed in a squamous carcinoma cell line (A431 cells) following activation with lysophosphatidic acid. The activation induced β-catenin translocation to the perinuclear endocytic recycling compartment that stained positive for RAB11A, a known marker for these structures [40]. RAB11A is associated primarily with recycling of endocytosed proteins and regulation of secretory pathways. Recycling endosomes are often located near the nucleus or in the centrosome, and are consequently referred to as perinuclear or pericentrosomal recycling endosomes [42]. We hypothesized that the accumulation of β-catenin trigger a regulatory process that enhances the compartmentalization and recycling of the latter.

### DNA sequencing of the MCR region and hot spot regions of the APC gene

**Fig. 6:**
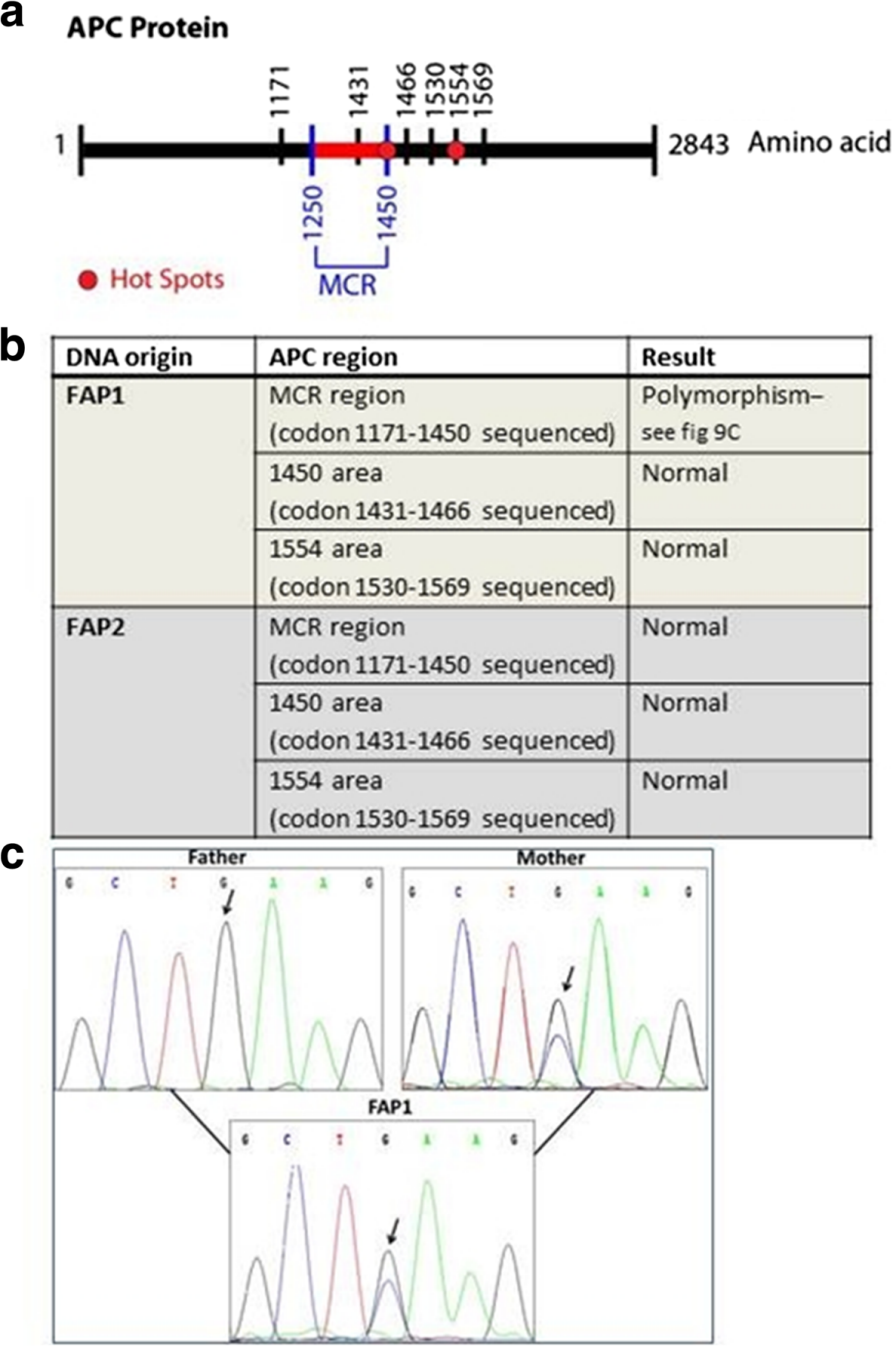
APC protein and sequencing chromatography results. **a** The APC protein scheme with the MCR and hot spot regions marked in *red* and sequenced area marked in *black*. **b** Sequencing results of the APC gene and the hot spots in genomic DNA extracted from FAP1 and FAP2 hESC lines at different passages. **c** Sequencing of codon 1317 region in the MCR of genomic DNA extracted from FAP1 cells at high passage (p53) as well as from both of the embryo donors (both maternal and paternal DNA). The mother has one WT allele that is altered to C in the second allele. The embryo inherited the mutated C allele from her mother and the WT G allele from the father

At this stage we wanted to explore the relationship between the increased β-catenin levels observed following extended culture of FAP1 hESCs and APC mutation. It is well established that the levels of expression and subcellular localization of β-catenin is regulated by the β-catenin destruction complex that includes the APC protein. A mutation in APC will result in a nonfunctional protein product that will lead to increased β-catenin levels. In search of the somatic mutations following extended cultures of FAP1 hESCs, we sequenced the entire MCR region (codons 1250–1450) and the hot spot regions (codons 1450 and 1554) within the APC gene in the FAP1 hESC lines from high passages (Fig. 6a,b). We were able to identify one difference in the MCR region of FAP1 hESCs that resulted in a G to C substitution leading to an E1317Q mutation (glutamic acid to glutamine substitution). In order to verify whether the difference we had found was inherited (polymorphism) or a novel somatic mutation, DNA samples from both parents of the donated embryo used for FAP1 derivation were also examined (Fig. 6c). Sequencing analysis demonstrated that this G to C mutation (E1317Q) in the APC gene was inherited from the mother, in addition to the germline R332X mutation inherited from the father, thus representing a polymorphism rather than a somatic mutation. It is important to note that this E1317Q polymorphism had reportedly correlated with colorectal neoplasia [43]. Having not been able to identify any mutations in the MCR region or in the familiar hot spot regions, we sequenced all the APC coding regions (Table 1). The DNA from high passage FAP1 hESCs was purified and sent to high throughput sequencing (ProntoDiagnostics). The results demonstrated several known polymorphisms, as indicated by their 50 % coverage (inherited from either the father or the mother) or 100 % coverage (inherited from both), but no new mutations indicative of a somatic mutation in the APC gene were identified (Table 1). We therefore concluded that activation of Wnt signaling following extended culture of FAP1 hESCs is probably not a result of the loss of function of the APC gene.

**Table 1:** Sequences of the APC coding regions

Exon location	Position (a.a)	Nuc. change	Coverage	Amino acid change	Nuc. name	Mutation
E11	332	C > T (het)	51 %	R- > (STOP) 332	c.994C > T	Germline
E13	486	T- > C (het)	50 %	Y- > Y (486)	c.1458 T > C rs2229992	Polymorphism
E15	545	G- > A (het)	52 %	A- > A (545)	c.1635 G > Ars351771	Polymorphism
E17	1317	G- > C (het)	50 %	E- > Q (1317)	c.3949G > A rs42427	Polymorphism
E17	1493	G- > A (het)	48 %	T- > T (1493)	c.4479G > A rs41115	Polymorphism
E17	1678	G- > A (het)	51 %	G- > G (1678)	c.5034G > A rs42427	Polymorphism
E17	1755	GTCT- > ATCG (het)	49 %	AS- > AS (1755..1756)	c.5265_5268delins	Polymorphism
E17	1822	T- > A (homo)	100 %	V- > D (1822)	c.5465 T > A rs459552	Polymorphism
E17	1960	G- > A (het)	51 %	P- > P (1960)	c.5880G > A rs465899	Polymorphism
E17	2401	C- > T (het)	53 %	L- > L (2401)	c.7201C > T rs2229994	Polymorphism

E – exone, het – heterozygote, homo - homozygote, Nuc - nucleotide

Another explanation for the differences between FAP1 and FAP2 hESCs may be that mutations in other components of the Wnt cascade, such as in Axin1 or GSK-3β, may have contributed to the high levels of Wnt signaling-mediated transcription in FAP1 hESCs but have not occurred in FAP2 hESC. It has been shown that mice that express an APC protein which lacks the armadillo repeat region, develop significantly more polyps than mice that express this region, very similar to the differences we observed between FAP1 and FAP2 [44]. The molecular mechanism behind this increased tumorigenesis remains unknown and we speculate that this may be due to the fact that the armadillo repeat region is a protein binding domain that binds a large number of proteins which may affect signaling pathways and tumorigenesis [45].

Another explanation for the increased β-catenin activity in FAP1 hESCs may be the differentiation state of the cells. Wnt/β-catenin signaling was shown to maintain self-renewal under feeder-free conditions in undifferentiated hESCs [24] while others have reported that Wnt signaling is involved in the differentiation of hESCs towards various lineages [36]. In the current study, we showed that Wnt /β-catenin signaling is low in undifferentiated FAP hESCs and that it is activated in extended culture of FAP1 hESCs. Our results on the undifferentiation state are inconsistent with those of Kathryn et al. [36] who showed negligible endogenous β-catenin signaling in undifferentiated hESCs. However, since activation of Wnt signaling is also linked to cell differentiation, it is possible that Wnt activation in our system reflects differentiation of at least some of the cells in culture, although the expression of pluripotent markers was >95 % in the undifferentiated cells. Interestingly, β-catenin was not activated in the FAP2 hESC line, demonstrating the diversity of the PGD-derived hESC lines that mimics the natural diversity of the human FAP population.

## Conclusions

The current study describes the establishment of FAP hESCs that carry the germline mutation in the APC gene as a novel human in-vitro model that can be used for studying the first steps in cancer development. To the best of our knowledge, this is the first report on PGD-derived hESC lines that carry mutation in a gene with predisposition for cancer [46]. Human pluripotent stem cells that carry tumor-associated mutations were recently shown to be extremely valuable for our understanding of pathological mechanisms involved in the development of different cancer types [47, 48]. FAP patients have an inherited germline mutation in one allele of the APC gene and loss or mutations of the second allele, leading to the development of polyps that will turn malignant if not removed. Thus, establishing a human-based in-vitro model system of FAP will enable us to study the early molecular mechanisms underlying tumorigenesis transformation in general and CRC development in particular. To this end, we derived two FAP hESC lines that were fully characterized as expressing key pluripotent markers and shown by karyotype analysis to be normal diploid. Confirmation of the germline mutation of the established cell lines demonstrated that they inherited the parental mutated APC allele. Genetic and epigenetic instability have been strongly associated with various types of cancer. Extended culture of hESCs has already been shown to be associated with genetic instability. We therefore hypothesized that during extended culture, the hESCs will acquire additional mutations, some of them in the wild type APC allele, which will result in complete loss of APC function and provide the cells with a selective growth advantage that will eventually dominate the entire population. Our results demonstrated that β-catenin/TCF-mediated activity is significantly increased following extended culture of FAP1-hESC cells. In accordance, β-catenin was translocated from the membrane to perinuclear structures. However, sequencing of the entire APC gene didn’t show the acquisition of a somatic mutation in these cells. We therefore concluded that activation of Wnt signaling following extended culture of FAP1-hESCs may not always be a result of APC loss of function, but rather may be due to mutations in other components of the Wnt cascade, such as in Axin1 or GSK-3β. Another explanation for the observed changes in β-catenin activity may be the differentiation state of the hESCs. It is possible that Wnt activation in our system reflects differentiation of at least some of the cells in culture. Interestingly, β-catenin was not activated in the FAP2 hESC line, demonstrating the diversity of the PGD-derived hESC lines that mimics the natural diversity of the human FAP population.

Taken together, these results demonstrate that our FAP-ESC lines are a unique human in vitro model with a mutation predisposition for cancer that can serve to study the early molecular and cellular events leading from such a single mutation into colorectal cancer.

In the cancer literature several other mutations like TP53 were also shown to cause a *predisposition* for *cancer*. TP53 function is frequently compromised during tumorigenesis as a result of homozygous somatic mutations, which are seen in more than 50 % of human cancers [47]. Single-copy mutation in TP53 has been implicated as the driver mutation for chemotherapy failure and poor prognosis in several specific cancers ([48–51]. Amir 2015 have recently shown that mutant clones of hESCs containing a single-copy deletion of TP53 exhibit significantly increased proliferation, enhanced colony formation and decreased levels of apoptosis upon exposure to chemotherapy drugs [52]. This study show that similarly, single-copy mutation that is present in the FAP1-hESCs may be sufficient for initiating cellular changes associated with tumorogenesis.

Using CRISPR/Cas9 genome engineering we are currently inducing mutations in the second copy of the APC gene as found in human CRC, mimicking normal progression of the disease. Using these innovative approaches with the FAP-hESCs studies here, will enable us to study in vitro, in a human based model, the earliest cellular and molecular events directly caused by the inactivation of APC and how this might lead to cancer initiation in otherwise normal cells. Furthermore, the development of an in vitro model system to study CRC from its earliest stages onward could greatly accelerate efforts toward the development of therapeutics and provide a platform for testing preventative strategies.

## Additional files

Additional file 1: Figure S1. (corresponding to Fig. 2). Confirmation of the APC germline mutation in FAP2 hESCs. (**A**) Amplification of the IVS14 + 1 G > A germline mutation area (178 bp) in the FAP2 line and in the WT hESC line. The BstNI differs between the WT allele (cut into 155 bp lengths) and the mutated allele (uncut 178 bp). (**B**) The mutation (IVS14 + 1 G > A) and 6 polymorphic markers flanking the mutation area D5S2051 and another five markers designed by us (M11, M1, M4, M14, M15) are shown in FAP2 hESCs that inherited the mutated APC allele from the affected father (mutated allele in red, normal alleles in black). (TIF 1990 kb)
Additional file 2: Figure S2. (corresponding to Fig. 3). Characterization of the FAP2 hESC line. (**A**) A colony of FAP2 cells at p29 with a typical morphology of hESCs. (**B**) Karyotype analysis. (**C**) FACS analysis for the pluripotent marker SSEA3. (**D**-**F**) Immunostaining for the pluripotent markers (green) Tra-1-60 (**D**), SSEA-4 (**E**), OCT4 (**F**) and the nuclear marker DAPI (blue) and their overlay. (TIF 1597 kb)
Additional file 3: Figure S3. Western blot analysis of β-catenin in FAP and in normal APC hESC lines. (**A**) Western blot analysis of β-catenin expression in FAP1 early (p12), FAP1 high (p82), FAP2 high (p80), HEFX1 high (p45), HUES64 high (p48) and HUES6 high (p53). β-actin served as a loading control. A representative gel is shown. (**B**) Densitometry analysis of three different western blotting experiments compared to the β-actin control using the "image J" software. All data were normalized to values obtained for the HUES6 line. (TIF 1800 kb)

## Abbreviations

APC: Adenomatous polyposis coli
CRC: Colorectal cancer
DAPI: 4’,6-diamidino-2-phenylindole
FAP: Familial adenomatous polyposis
hESCs: Human embryonic stem cells
ICM: Inner cell mass
IVF: In-Vitro fertilization
LEF: Lymphoid enhancer factor
MCR: Mutation cluster region
MEFs: Mouse embryonic fibroblast cells
PBS: Phosphate buffered solution
PBT: PBS containing 0.1 % Triton
PGD: Preimplantation genetic diagnosis
SDS-PAGE: SDS-Polyacrylamide gel electrophoresis
TCF: T-Cell factor
WT: Wild-type

## References

[1] Jemal A, Siegel R, Ward E, Murray T, Xu J, Thun MJ (2007). Cancer statistics, 2007. CA Cancer J Clin.

[2] Giles RH, van Es JH, Clevers H (2003). Caught up in a Wnt storm: Wnt signaling in cancer. Biochim Biophys Acta.

[3] Groden J, Thliveris A, Samowitz W, Carlson M, Gelbert L, Albertsen H, Joslyn G, Stevens J, Spirio L, Robertson M (1991). Identification and characterization of the familial adenomatous polyposis coli gene. Cell.

[4] Aoki K, Taketo MM (2007). Adenomatous polyposis coli (APC): a multi-functional tumor suppressor gene. J Cell Sci.

[5] van Es JH, Giles RH, Clevers HC (2001). The many faces of the tumor suppressor gene APC. Exp Cell Res.

[6] Skalka N, Caspi M, Caspi E, Loh YP, Rosin-Arbesfeld R. Carboxypeptidase E: a negative regulator of the canonical Wnt signaling pathway. Oncogene. 2012. 10.1038/onc.2012.308 PMC3676431 22824791

[7] Kinzler KW, Nilbert MC, Su LK, Vogelstein B, Bryan TM, Levy DB, Smith KJ, Preisinger AC, Hedge P, McKechnie D (1991). Identification of FAP locus genes from chromosome 5q21. Science.

[8] Nishisho I, Nakamura Y, Miyoshi Y, Miki Y, Ando H, Horii A, Koyama K, Utsunomiya J, Baba S, Hedge P (1991). Mutations of chromosome 5q21 genes in FAP and colorectal cancer patients. Science.

[9] Lamlum H, Ilyas M, Rowan A, Clark S, Johnson V, Bell J, Frayling I, Efstathiou J, Pack K, Payne S (1999). The type of somatic mutation at APC in familial adenomatous polyposis is determined by the site of the germline mutation: a new facet to Knudson's 'two-hit' hypothesis. Nat Med.

[10] Albuquerque C, Breukel C, van der Luijt R, Fidalgo P, Lage P, Slors FJ, Leitao CN, Fodde R, Smits R (2002). The 'just-right' signaling model: APC somatic mutations are selected based on a specific level of activation of the beta-catenin signaling cascade. Hum Mol Genet.

[11] Burt RW, Leppert MF, Slattery ML, Samowitz WS, Spirio LN, Kerber RA, Kuwada SK, Neklason DW, Disario JA, Lyon E (2004). Genetic testing and phenotype in a large kindred with attenuated familial adenomatous polyposis. Gastroenterology.

[12] Nieuwenhuis MH, Vasen HF (2007). Correlations between mutation site in APC and phenotype of familial adenomatous polyposis (FAP): a review of the literature. Crit Rev Oncol Hematol.

[13] Rowan AJ, Lamlum H, Ilyas M, Wheeler J, Straub J, Papadopoulou A, Bicknell D, Bodmer WF, Tomlinson IP (2000). APC mutations in sporadic colorectal tumors: A mutational "hotspot" and interdependence of the "two hits". Proc Natl Acad Sci U S A.

[14] Smits R, Hofland N, Edelmann W, Geugien M, Jagmohan-Changur S, Albuquerque C, Breukel C, Kucherlapati R, Kielman MF, Fodde R (2000). Somatic Apc mutations are selected upon their capacity to inactivate the beta-catenin downregulating activity. Genes Chromosomes Cancer.

[15] van der Luijt RB, Vasen HF, Tops CM, Breukel C, Fodde R, Meera Khan P (1995). APC mutation in the alternatively spliced region of exon 9 associated with late onset familial adenomatous polyposis. Hum Genet.

[16] Walon C, Kartheuser A, Michils G, Smaers M, Lannoy N, Ngounou P, Mertens G, Verellen-Dumoulin C (1997). Novel germline mutations in the APC gene and their phenotypic spectrum in familial adenomatous polyposis kindreds. Hum Genet.

[17] Ben-Yehudah A, Malcov M, Frumkin T, Ben-Yosef D (2012). Mutated human embryonic stem cells for the study of human genetic disorders. Methods Mol Biol.

[18] Heyer J, Yang K, Lipkin M, Edelmann W, Kucherlapati R (1999). Mouse models for colorectal cancer. Oncogene.

[19] Taketo MM, Edelmann W (2009). Mouse models of colon cancer. Gastroenterology.

[20] Nandan MO, Yang VW (2010). Genetic and Chemical Models of Colorectal Cancer in Mice. Curr Color Cancer Rep.

[21] Telias M, Segal M, Ben-Yosef D (2013). Neural differentiation of Fragile X human Embryonic Stem Cells reveals abnormal patterns of development despite successful neurogenesis. Dev Biol.

[22] Eiges R, Urbach A, Malcov M, Frumkin T, Schwartz T, Amit A, Yaron Y, Eden A, Yanuka O, Benvenisty N (2007). Developmental study of fragile X syndrome using human embryonic stem cells derived from preimplantation genetically diagnosed embryos. Cell Stem Cell.

[23] Frumkin T, Malcov M, Yaron Y, Ben-Yosef D (2008). Elucidating the origin of chromosomal aberrations in IVF embryos by preimplantation genetic analysis. Mol Cell Endocrinol.

[24] Sato N, Meijer L, Skaltsounis L, Greengard P, Brivanlou AH (2004). Maintenance of pluripotency in human and mouse embryonic stem cells through activation of Wnt signaling by a pharmacological GSK-3-specific inhibitor. Nat Med.

[25] Ben-Yosef D, Boscolo FS, Amir H, Malcov M, Amit A, Laurent LC (2013). Genomic analysis of hESC pedigrees identifies de novo mutations and enables determination of the timing and origin of mutational events. Cell Rep.

[26] Ben-Yosef D, Amit A, Malcov M, Frumkin T, Ben-Yehudah A, Eldar I, Mey-Raz N, Azem F, Altarescu G, Renbaum P (2011). Female sex bias in human embryonic stem cell lines. Stem Cells Dev.

[27] Frumkin T, Malcov M, Telias M, Gold V, Schwartz T, Azem F, Amit A, Yaron Y, Ben-Yosef D (2010). Human embryonic stem cells carrying mutations for severe genetic disorders. In Vitro Cell Dev Biol Anim.

[28] Eiges R, Schuldiner M, Drukker M, Yanuka O, Itskovitz-Eldor J, Benvenisty N (2001). Establishment of human embryonic stem cell-transfected clones carrying a marker for undifferentiated cells. Curr Biol.

[29] Bock C, Kiskinis E, Verstappen G, Gu H, Boulting G, Smith ZD, Ziller M, Croft GF, Amoroso MW, Oakley DH (2011). Reference Maps of human ES and iPS cell variation enable high-throughput characterization of pluripotent cell lines. Cell.

[30] Cowan CA, Klimanskaya I, McMahon J, Atienza J, Witmyer J, Zucker JP, Wang S, Morton CC, McMahon AP, Powers D (2004). Derivation of embryonic stem-cell lines from human blastocysts. N Engl J Med.

[31] Osafune K, Caron L, Borowiak M, Martinez RJ, Fitz-Gerald CS, Sato Y, Cowan CA, Chien KR, Melton DA (2008). Marked differences in differentiation propensity among human embryonic stem cell lines. Nat Biotechnol.

[32] Malcov M, Naiman T, Yosef DB, Carmon A, Mey-Raz N, Amit A, Vagman I, Yaron Y (2007). Preimplantation genetic diagnosis for fragile X syndrome using multiplex nested PCR. Reprod BioMed Online.

[33] Dvash T, Mayshar Y, Darr H, McElhaney M, Barker D, Yanuka O, Kotkow KJ, Rubin LL, Benvenisty N, Eiges R (2004). Temporal gene expression during differentiation of human embryonic stem cells and embryoid bodies. Hum Reprod.

[34] Amps K, Andrews PW, Anyfantis G, Armstrong L, Avery S, Baharvand H, Baker J, Baker D, Munoz MB, Beil S (2011). Screening ethnically diverse human embryonic stem cells identifies a chromosome 20 minimal amplicon conferring growth advantage. Nat Biotechnol.

[35] Avery S, Hirst AJ, Baker D, Lim CY, Alagaratnam S, Skotheim RI, Lothe RA, Pera MF, Colman A, Robson P (2013). BCL-XL Mediates the Strong Selective Advantage of a 20q11.21 Amplification Commonly Found in Human Embryonic Stem Cell Cultures. Stem Cell Rep.

[36] Davidson KC, Adams AM, Goodson JM, McDonald CE, Potter JC, Berndt JD, Biechele TL, Taylor RJ, Moon RT (2012). Wnt/beta-catenin signaling promotes differentiation, not self-renewal, of human embryonic stem cells and is repressed by Oct4. Proc Natl Acad Sci U S A.

[37] Draper JS, Smith K, Gokhale P, Moore HD, Maltby E, Johnson J, Meisner L, Zwaka TP, Thomson JA, Andrews PW (2004). Recurrent gain of chromosomes 17q and 12 in cultured human embryonic stem cells. Nat Biotechnol.

[38] Mitalipova MM, Rao RR, Hoyer DM, Johnson JA, Meisner LF, Jones KL, Dalton S, Stice SL (2005). Preserving the genetic integrity of human embryonic stem cells. Nat Biotechnol.

[39] Logan CY, Nusse R (2004). The Wnt signaling pathway in development and disease. Annu Rev Cell Dev Biol.

[40] Kam Y, Quaranta V (2009). Cadherin-bound beta-catenin feeds into the Wnt pathway upon adherens junctions dissociation: evidence for an intersection between beta-catenin pools. PLoS One.

[41] Krieghoff E, Behrens J, Mayr B (2006). Nucleo-cytoplasmic distribution of beta-catenin is regulated by retention. J Cell Sci.

[42] Takahashi S, Kubo K, Waguri S, Yabashi A, Shin HW, Katoh Y, Nakayama K (2012). Rab11 regulates exocytosis of recycling vesicles at the plasma membrane. J Cell Sci.

[43] Hall MJ, Liberman E, Dulkart O, Galazan L, Sagiv E, Shmueli E, Kazanov D, Hallak A, Moshkowitz M, Figer A (2009). Risk of colorectal neoplasia associated with the adenomatous polyposis coli E1317Q variant. Ann Oncol.

[44] Crist RC, Roth JJ, Baran AA, McEntee BJ, Siracusa LD, Buchberg AM (2010). The armadillo repeat domain of Apc suppresses intestinal tumorigenesis. Mam Genome.

[45] Morishita EC, Murayama K, Kato-Murayama M, Ishizuka-Katsura Y, Tomabechi Y, Hayashi T, Terada T, Handa N, Shirouzu M, Akiyama T (2011). Crystal structures of the armadillo repeat domain of adenomatous polyposis coli and its complex with the tyrosine-rich domain of Sam68. Structure.

[46] Verlinsky Y, Strelchenko N, Kukharenko V, Rechitsky S, Verlinsky O, Galat V, Kuliev A (2005). Human embryonic stem cell lines with genetic disorders. Reprod BioMed Online.

[47] Brosh R, Rotter V (2009). When mutants gain new powers: news from the mutant p53 field. Nat Rev Cancer.

[48] Shindiapina P, Brown JR, Danilov AV (2014). A new hope: novel therapeutic approaches to treatment of chronic lymphocytic leukaemia with defects in TP53. Br J Haematol.

[49] Puiggros A, Blanco G, Espinet B (2014). Genetic abnormalities in chronic lymphocytic leukemia: where we are and where we go. BioMed Res Int.

[50] Halldorsdottir AM, Lundin A, Murray F, Mansouri L, Knuutila S, Sundstrom C, Laurell A, Ehrencrona H, Sander B, Rosenquist R (2011). Impact of TP53 mutation and 17p deletion in mantle cell lymphoma. Leukemia.

[51] Teoh PJ, Chung TH, Sebastian S, Choo SN, Yan J, Ng SB, Fonseca R, Chng WJ (2014). p53 haploinsufficiency and functional abnormalities in multiple myeloma. Leukemia.

[52] Amir H (2015). Spontaneous Single-Copy Deletion of chr17p13.1 in Human Embryonic Stem Cells Improves Cell Survival By Decreasing Expression of TP53. Oral presentation.

