# Supplementary figures and images for: The effect of a germline mutation in the APC gene on β-catenin in human embryonic stem cells
Source: BMC Cancer. 2016 Dec 23;16:952. doi: 10.1186/s12885-016-2809-9 (PMC5180406; doi:10.1186/s12885-016-2809-9)

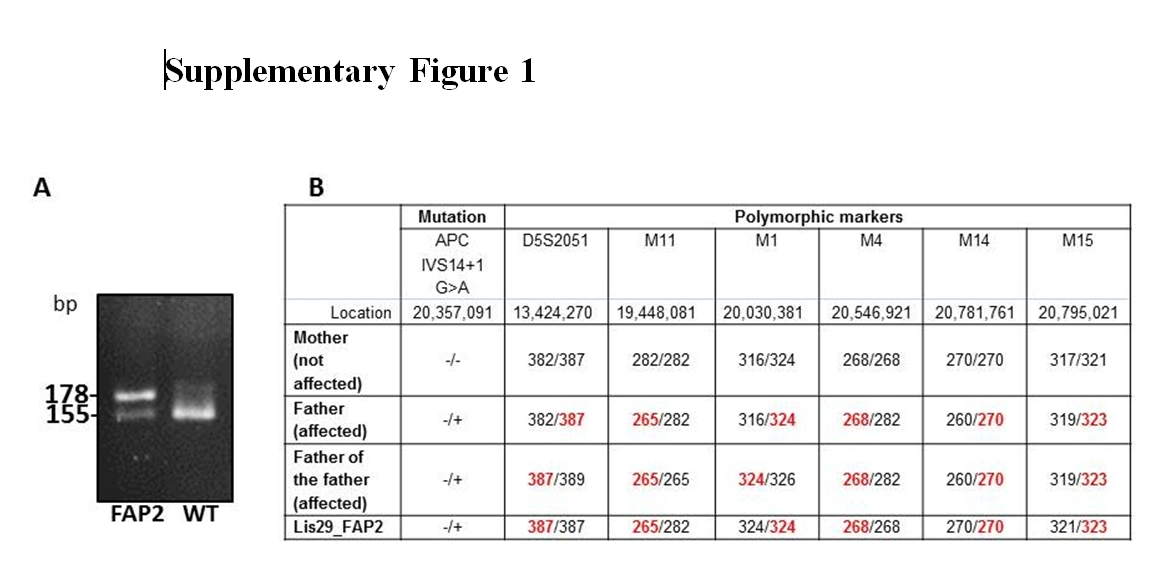

Supplement: Additional file 1: Figure S1. — (corresponding to Fig. 2). Confirmation of the APC germline mutation in FAP2 hESCs. (A) Amplification of the IVS14 + 1 G > A germline mutation area (178 bp) in the FAP2 line and in the WT hESC line. The BstNI differs between the WT allele (cut into 155 bp lengths) and the mutated allele (uncut 178 bp). (B) The mutation (IVS14 + 1 G > A) and 6 polymorphic markers flanking the mutation area D5S2051 and another five markers designed by us (M11, M1, M4, M14, M15) are shown in FAP2 hESCs that inherited the mutated APC allele from the affected father (mutated allele in red, normal alleles in black). (TIF 1990 kb) [file 12885_2016_2809_MOESM1_ESM.tif]

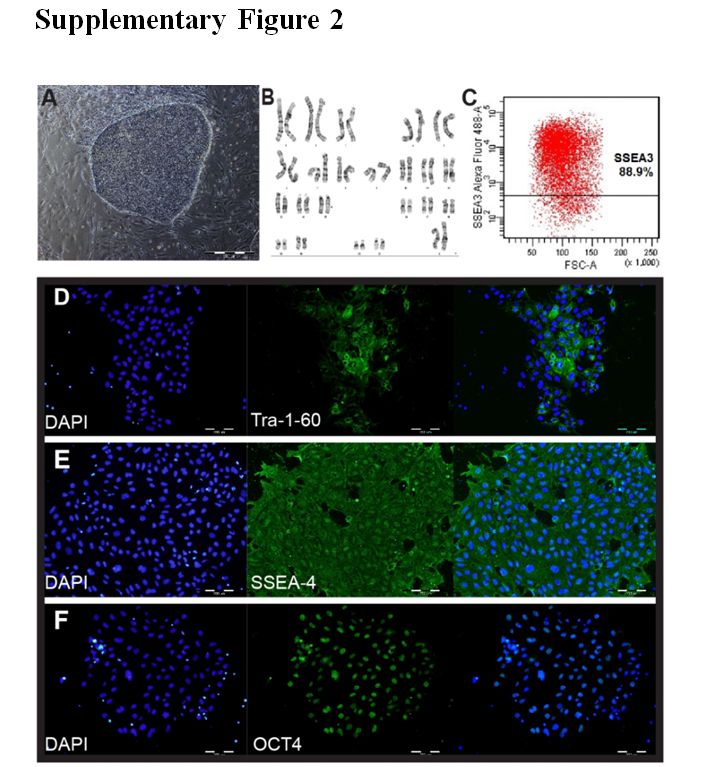

Supplement: Additional file 2: Figure S2. — (corresponding to Fig. 3). Characterization of the FAP2 hESC line. (A) A colony of FAP2 cells at p29 with a typical morphology of hESCs. (B) Karyotype analysis. (C) FACS analysis for the pluripotent marker SSEA3. (D-F) Immunostaining for the pluripotent markers (green) Tra-1-60 (D), SSEA-4 (E), OCT4 (F) and the nuclear marker DAPI (blue) and their overlay. (TIF 1597 kb) [file 12885_2016_2809_MOESM2_ESM.tif]

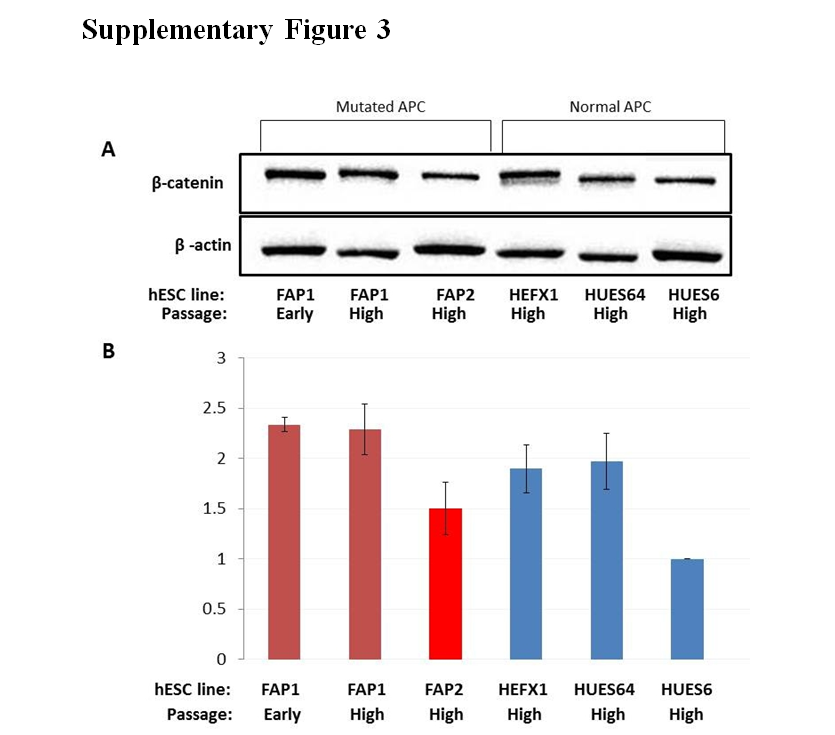

Supplement: Additional file 3: Figure S3. — Western blot analysis of β-catenin in FAP and in normal APC hESC lines. (A) Western blot analysis of β-catenin expression in FAP1 early (p12), FAP1 high (p82), FAP2 high (p80), HEFX1 high (p45), HUES64 high (p48) and HUES6 high (p53). β-actin served as a loading control. A representative gel is shown. (B) Densitometry analysis of three different western blotting experiments compared to the β-actin control using the "image J" software. All data were normalized to values obtained for the HUES6 line. (TIF 1800 kb) [file 12885_2016_2809_MOESM3_ESM.tif]
